# Development of a Radiolabeled Peptide-Based Probe Targeting MT1-MMP for Breast Cancer Detection

**DOI:** 10.1371/journal.pone.0139471

**Published:** 2015-10-05

**Authors:** Kaiyin Min, Bin Ji, Min Zhao, Tiefeng Ji, Bin Chen, Xuedong Fang, Qingjie Ma

**Affiliations:** 1 Department of Nuclear Medicine, China-Japan Union Hospital of Jilin University, Changchun 130033, China; 2 Department of General Surgery, China-Japan Union Hospital of Jilin University, Changchun 130033, China; The University of Hong Kong, HONG KONG

## Abstract

Breast cancer is one of the most frequent and aggressive primary tumors among women of all races. Matrix metalloproteinase (MMPs), a family of zinc- and calcium-dependent secreted or membrane anchored endopeptidases, is overexpressed in varieties of diseases including breast cancer. Therefore, noninvasive visualization and quantification of MMP *in vivo* are of great interest in basic research and clinical application for breast cancer early diagnosis. Herein, we developed a ^99m^Tc labeled membrane type I matrix metalloproteinase (MT1-MMP) specific binding peptide, [^99m^Tc]-(HYNIC-AF7p)(tricine)(TPPTS), for *in vivo* detection of MDA-MB-231 breast tumor by single photon emission computed tomography (SPECT). [^99m^Tc]-(HYNIC-AF7p)(tricine)(TPPTS) demonstrated nice biostability and high MT1-MMP binding affinity *in vitro* and *in vivo*. Tumor-to-muscle ratio was found to reach to the highest (4.17±0.49) at 2 hour after intravenously administration of [^99m^Tc]-(HYNIC-AF7P)(tricine)(TPPTS) into MDA-MB-231 tumor bearing mice. Overall, [^99m^Tc]-(HYNIC-AF7P)(tricine)(TPPTS) demonstrated great potential for MT1-MMP targeted detection *in vivo* and it would be a promising molecular imaging probe that are probably beneficial to breast cancer early diagnoses.

## Introduction

Breast cancer is one of the leading causes of cancer death among women of all races. The life time risky for individuals to suffer breast cancer could be as high as 13%[1, 2]. Accurate diagnosis of breast cancer is very important and desirable, by which various therapeutic regimens could be given before the primary tumors become metastatic.

Matrix metalloproteinases (MMPs) are a family of zinc-dependent endopeptidases that degrade most of extra-cellular matrix (ECM) proteins. It plays an important role in the development of various diseases including cancer [3], inflammation, neurological and cardiovascular diseases. In the case of cancer, MMPs not only have distinct roles in tumor angiogenesis, but also affect multiple signaling pathways to control the balance between growth and antigrowth signals in the tumor microenvironment. Membrane type-1 matrix metalloproteinase (MT1-MMP) has been shown to be a key member of the MMP family with much biological and pathological significance [4, 5]. Specifically, MT1-MMP is intrinsically associated with the plasma membrane of normal and tumor cells and remodels the extracellular matrix (ECM). In patients with breast cancer, MT1-MMP has been reported with high expression in both serum and local lesions [6, 7]. Although the failures of applications of MMP inhibitors in tumor prevention and therapy have been reported [8, 9], there are accumulating reports about the potential of utilization MMP as target for cancer diagnosis [10–12].

So far, MT1-MMP targeting peptides for breast cancer imaging and therapy are being widely studied in both experimental and clinical settings [13–16]. The first reason of development of small molecules capable to track MT1-MMP *in vivo* is to help investigating the nature of MT1-MMP expression, distribution, and its biological and pathological functions. Secondly, a specific MT1-MMP targeting molecule would also provide the chance of earlier detection and characterization of disease and evaluation of treatment in diseases where MT1-MMP is overexpressed. Recently, we have developed a high MT1-MMP affinity peptide, named AF7p, by phage display peptide library screening technique. MT1-MMP high expressed tumor was successfully imaged *in vivo* by a near infrared dye labeled AF7p, suggesting the potential of utilizing AF7p for MT1-MMP targeted tumor detection [12]. Compared with optical imaging, radioisotope mediated imaging is more sensitive and accurate in clinic with less tissue penetration and quantification limitation. Currently both single photon emissions computed tomography (SPECT) and positron emission tomography (PET) are well developed [17–19]. Generally, PET imaging holds great promise in visualization of biology activities, but to some extent the short half-life of positron emitting radionuclide, high cost of instruments and the complicated preparation of tracers slow down the application of PET in clinic. On the other hand, SPECT can offer a simple and inexpensive procedure with readily available ^99m^Tc through inexpensive ^99^Mo-^99m^Tc generator [20–22]. Overall, SPECT is more likely to be a cost-effective technique in early detection of breast cancer in clinical practice [23].

In the present study, to develop an SPECT probe for breast cancer early diagnosis, we modified AF7p with 6-hydrazinonicotinic acid (HYNIC) as the biomolecule for preparing a ^99m^Tc-labeled MT1-MMP targeted imaging agent. The preparation, stability, biodistribution and tumor tagetability of [^99m^Tc]-(HYNIC-AF7p)(tricine)(TPPTS) was presented and evaluated. Our results suggested that [^99m^Tc]-(HYNIC-AF7p) is promising for MT1-MMP overexpressed tumor detection with high affinity and specificity.

## Materials and Methods

### Reagents

Chemicals were purchased from Sigma-Aldrich (USA) and were used without further purification. Side chain protected peptide with the sequence of His-Trp-Lys(Dde)-His-Leu-His-Asn-Thr-Lys(Dde)-Thr-Phe-Leu was custom-made by the GL Biochem, Ltd. (Shanghai, China). Na^99m^TcO4 was obtained from a commercial ^99^Mo/^99m^Tc generator (Beijing Atom High Tech Co., Ltd.).

### Synthesis of HYNIC-conjugated AF7P

The HYNIC-AF7p conjugate was prepared by coupling AF7p with HYNIC-NHS in slightly basic condition (pH 8.5) in DMF [24, 25]. Briefly, NHS-HYNIC (216.03 μg, 0.8641 μmol) and AF7p-Dde (1.23 mg, 0.6043 μmol) were dissolved in DMF (200 μL) and DIPEA (0.43 μL, 2.63 μmol) was further added to adjust pH around 8 to 8.5. The mixture was stirred for 10 h at room temperature. After dried under vacuum, the HYNIC-AF7p-Dde peptide was dissolved in 190 μL DMF. The Dde-group was removed by treating HYNIC-AF7p-Dde with 5% hydrazine (10 μL) for 40 min at room temperature. The product was then isolated and purified by HPLC. The fraction at retention time of 11.93 min was collected. Lyophilization of the combined collections afforded the expected product, HYNIC-AF7P. Matrix-assisted laser desorption/ionization (MALDI) time-of-light (TOF) mass spectral data of HYNIC-AF7p was m/z = 1839.61 for [M + H]+ (C87H125N25O20, calculated molecular weight 1839.95 Da). The purity of all the final products were confirmed by analytical HPLC, using 5% to 65% acetonitrile containing 0.1% TFA versus distilled water containing 0.1% TFA over 30 minutes at a flow rate of 1 mL/min (C18 column, 5 μm, 120Å, 250 × 4.6 mm) and purity > 95%.

### Synthesis of [^99m^Tc]-(HYNIC-AF7p) (tricine) (TPPTS)

HYNIC-AF7p solution (20 μL, 60 μg/mL in H_2_O), of tricine solution (100 μL, 100 mg/mL in 25 mM succinate buffer, pH 5.0), SnCl_2_ solution (10 μL, 3 mg/mL in 0.1 M HCl) and Na^99m^TcO_4_ (100 μL, 370 MBq) in saline were added into a 5 mL Eppendorf tube. The reaction mixture was kept at room temperature for 10 min. To the reaction mixture above was added 100 μL of the TPPTS solution (50 mg/mL in 25 mM succinate buffer, pH 5.0). The vial containing the reaction mixture was sealed, cramped, and heated at 90°C for 30 min. After cooling to room temperature, sample was analyzed by radio-HPLC. The radio-HPLC method used a HP Hewlett Packard Series 1100 HPLC system equipped with a Radioflow Detector LB509 and a reversed-phase Zorbax SB-C18 column (4.6 mm × 250 mm, 5 μm). The flow rate was 1 mL/min. The gradient mobile phase started with 90% solvent A (0.1% TFA in water) and 10% solvent B (0.1% TFA in acetonitrile) to 30% solvent A and 70% solvent B at 24 min to 0% solvent A and 100% solvent B at 27 min, followed with 90% solvent A and 10% solvent B at 30 min. Gelman Sciences silica-gel paper strips was used in ITLC method. A 1:1 mixture of acetone and saline were used as eluent. The ^99m^Tc complexes migrated with the solvent front (Rf = 1.0) while ^99m^TcO_4_ and ^99m^Tc-AF7p remained at origin (Rf = 0.0).

### *In vitro* stability of [^99m^Tc]-(HYNIC-AF7p) (tricine) (TPPTS)

100 μL of [^99m^Tc]-(HYNIC-AF7P)(tricine)(TPPTS) was added to either 0.9% physiological saline or 1 mg/mL cysteine solution. The total volume was adjusted to 400 μL. The mixed solution was incubated at 37°C. After 1 h, 2 h, 3 h, 4 h, 5 h and 6 h incubation, the radiochemical purity for [^99m^Tc]-(HYNIC-AF7P)(tricine)(TPPTS) at each time point was detected by radio-HPLC as described above.

### Tumor immunohistochemistry

MDA-MB-435 and A549 tumor bearing mice were sacrificed and tumors were frozen in OCT embedding medium. Cryosections were cut into 4 μm and subjected to staining. Briefly, tumor slides were dried in the air and fixed with cold acetone for 20 min and dried again in the air for 30 min at room temperature. After blocking with 10% BSA for 30 min, the sections were incubated with rabbit anti-MT1-MMP antibody (10 μg/mL, Abcam, MA) for 60 min at room temperature in the dark, and then visualized with FITC-conjugated donkey anti-rabbit secondary antibody. Finally, the slices were mounted with DAPI-containing mounting medium under a fluorescence microscope (Olympus, X81). Fluorescence pictures were taken using FITC filter settings (excitation = 490 nm, emission = 520 nm).

### Fluorescent staining

The MDA-MB-231 and A549 tumor cells were cultured at 37°C and 5% CO_2_ in DMEM containing 10% fetal bovine serum (FBS). MDA-MB-231 and A549 cells were seeded into eight-well chamber at the concentration of 1×10^4^ cells/well for cell immunostaining. The next day, cells were fixed by 90% cold ether for 20 min at -20°C. After being blocked by 10% BSA at 37°C, MDA-MB-231 and A549 cells were detected using 2 μg/mL rabbit anti-MMP14 primary antibody for 2 h at room temperature, and then visualized by FITC conjugated donkey anti-rabbit secondary antibody (1: 100 dilution) together with 10 nM of Cy5.5 (ex/em: 675/695 nm) conjugated AF7p, Cy5.5-AF7p [12]. After washing steps, cells were mounted with 4′, 6-diamidino-2-phenylindole (DAPI)–containing mounting medium and observed with a fluorescence microscope. For blocking experiment, fixed MDA-MB-231 and A549 cells were seeded into another eight-well chamber at the concentration of 1×10^4^ cells/well and blocked with 10% BSA for 1 h. Next, 5 μM free AF7p was added into each well before Cy5.5-AF7p (10 nM) was added. After washing steps, cells were mounted with 4′, 6-diamidino-2-phenylindole (DAPI)–containing mounting medium and observed with a fluorescence microscope.

### *In vitro* cytotoxicity of AF7p analogs

A standard Cell Counting Kit-8 (CCK-8) was utilized to analyze the cytotoxicity of AF7p analogs following a general protocol. Briefly, MDA-MB-231 cells were seeded in a 96-well plate with the concentration of 5×10^4^ cells/well. After incubation at 37°C for 24 h, AF7p analogs with a final concentration of 10, 50, 100, 200, 500 or 1000 nM were incubated with cells for 24 h, after which 10 μL of CCK-8 solution was added to each well of the 96-well plate and incubated for another 4 h. The amount of an orange formazan dye, produced by the reduction of WST-8 (active gradient in CCK-8) by dehydrogenases in live cells, is directly proportional to the quantity of live cells in the well. Therefore, by measuring the absorbance of each well at 450 nm using a microplate reader, cell viability could be determined with the calculation of the ratio of absorbance of experimental well to that of the cell control well. All experiments were triplicated and results were averaged.

### Preparation of tumor-bearing animals

All animal experiments were approved by Jilin University Health Science Center Animal Care and Use Committee. Human breast carcinoma cell line MDA-MB-231 with high MT1-MMP expression and human lung adenocarcinomaepithelial cell line A549 with low MT1-MMP expression were cultured in DMEM medium containing 10% (v/v) fetal bovine serum (Invitrogen) supplemented with penicillin (100 μg/mL) and streptomycin (100 μg/mL) at 37°C with 5% CO2. MDA-MB-231 and A549 tumor models were established by subcutaneous injection of 5×106 cells into the upper right front flank of female athymic nude mice (Department of Experimental Animal, Peking University Health Science Center). Tumor growth was monitored by caliper measurements three times a week after the tumors are palpable. The mice were used for SPET/CT imaging when the tumor volume reached about 200 mm3 (about 14 days after tumor inoculations). The tumor volume was determined as the formula: V = a × (b^2^)/2, where a and b are the length and width of each tumor in mm respectively. During the injection and image acquisition process, the mice were anesthetized with 2.5% isoflurane in oxygen delivered at a flow rate of 1.5 L/min.

### Imaging study

The imaging study was performed using the female BALB/c nude mice bearing the MDA-MB-231 tumors and A549 tumors. Each tumor-bearing mouse was administered intravenously with [^99m^Tc]-(HYNIC-AF7P)(tricine)(TPPTS) (22.2 MBq/mice) in 0.1 mL saline. For blocking test, 200 μg free AF7P was intravenously injected into MDA-MB-231 tumor bearing mice 30 min before [^99m^Tc]-(HYNIC-AF7P)(tricine)(TPPTS) was injected. Animals were placed prone on a two-head γ-camera (SIEMENS, E. CAM) equipped with a parallel-hole, low-energy, and high-resolution collimator. Posterior images were acquired at 0.5, 2 and 4 hours post injection (p.i.). The acquisition count limits were set at 700k. After completion of imaging, animals were sacrificed by cervical dislocation.

### *In vivo* biodistribution study

Animals were divided into 4 groups (n = 3) for 4 time points with approximately equal distribution of tumor sizes on the day before the study. At 0.5, 1, 2, and 4 h after intravenous administration of [^99m^Tc]-(HYNIC-AF7P)(tricine)(TPPTS), MDA-MB-231 tumor bearing mice were euthanized. Blood, heart, lung, liver, kidney, stomach, intestine, spleen, pancreas, brain, muscle and tumor were excised, weighed and counted for radioactivity. The organ uptake was calculated as a percentage of the injected dose per gram of wet tissue (%ID/g).

### Statistical analysis

Results were expressed as mean ± SD. Two-tailed paired and unpaired Student’s t tests were used to test differences within groups and between groups, respectively. P values < 0.05 were considered statistically significant.

## Results

### Radiochemistry

**Fig 1:**
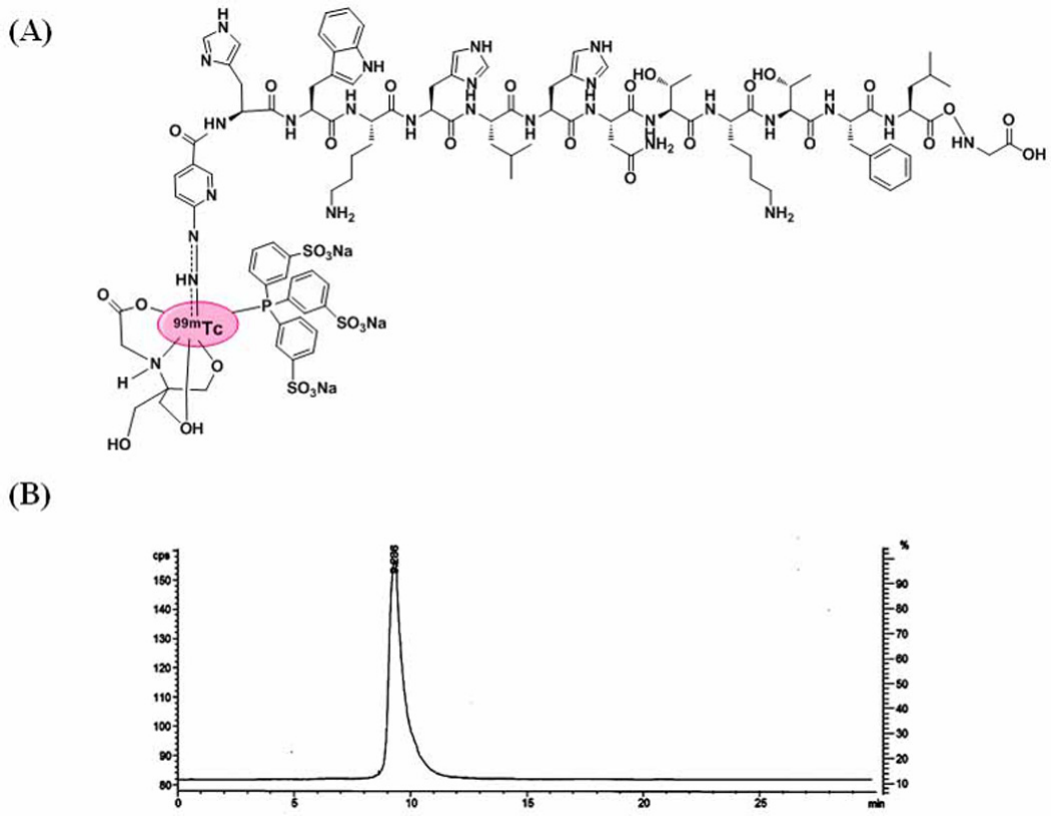
Characterizations. (A) Chemical structure of [^99m^Tc]-(HYNIC-AF7p)(tricine)(TPPTS). (B) Radio-HPLC purity of [^99m^Tc]-(HYNIC-AF7p)(tricine)(TPPTS).

[^99m^Tc]-(HYNIC-AF7P)(tricine)(TPPTS) was prepared in two steps. First, HYNIC-AF7P was synthesized (Fig 1) and reacted with Na^99m^TcO_4_ in the presence of excess tricine and SnCl_2_ to form the intermediate [^99m^Tc]-(HYNIC-AF7P)(tricine)_2_, which was then allowed to react with TPPTS to form the ternary ligand complex [^99m^Tc](HYNIC-AF7P)(tricine)(TPPTS). After Sep-Pak C_18_ column purification, the radiochemical purity was found to be above 95% determinated by analytical Radio-HPLC (Fig 1). The HPLC retention time was 9.2 min for [^99m^Tc](HYNIC-AF7P)(tricine)(TPPTS) and the specific activity of final product was 1200 mCi/μmol. The partition coefficient of [^99m^Tc]-(HYNIC-AF7P)(tricine)(TPPTS) was determined in an equal volume mixture of noctanoland phosphate buffer (25 mM, pH = 7.4). Log P value of [^99m^Tc]-(HYNIC-AF7P)(tricine)(TPPTS)was -2.39±0.06.

### Stability

**Fig 2:**
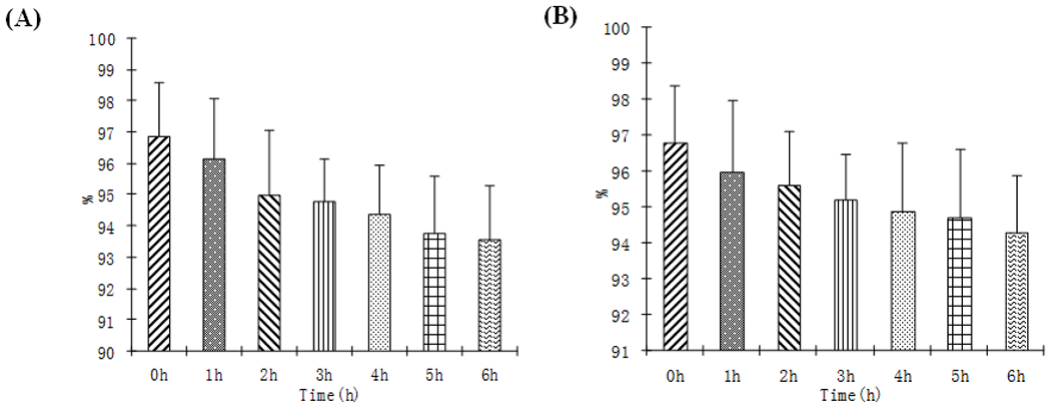
Solution stability data for [^99m^Tc(HYNIC-AF7P)(tricine)(TPPTS)] in saline (A) and in the presence of excess cysteine (B) (1 mg/mL, pH = 7.4).

For *in vivo* application, we have to make sure the probe keeps intact before it arrives at the tumor area. We then compared the *in vitro* stability of [^99m^Tc]-(HYNIC-AF7P)(tricine)(TPPTS) in saline and saline with excess amount of cysteine. As shown in Fig 2, during the 6 hours incubation, the original form of [^99m^Tc]-(HYNIC-AF7P)(tricine)(TPPTS) in saline only decreased slightly from 96.8 ± 1.12% at the beginning to 93.5 ± 2.27% (Fig 2A). Similarly, the amount decreased from 96.8 ± 1.52% to 94.2 ±1.72% in the solution containing excess cysteine during the 6 hours incubation (Fig 2B). It is quite clear that [^99m^Tc]-(HYNIC-AF7P)(tricine)(TPPTS) is able to maintain its stability over6 h in saline and in the presence of excess cysteine.

**Fig 3:**
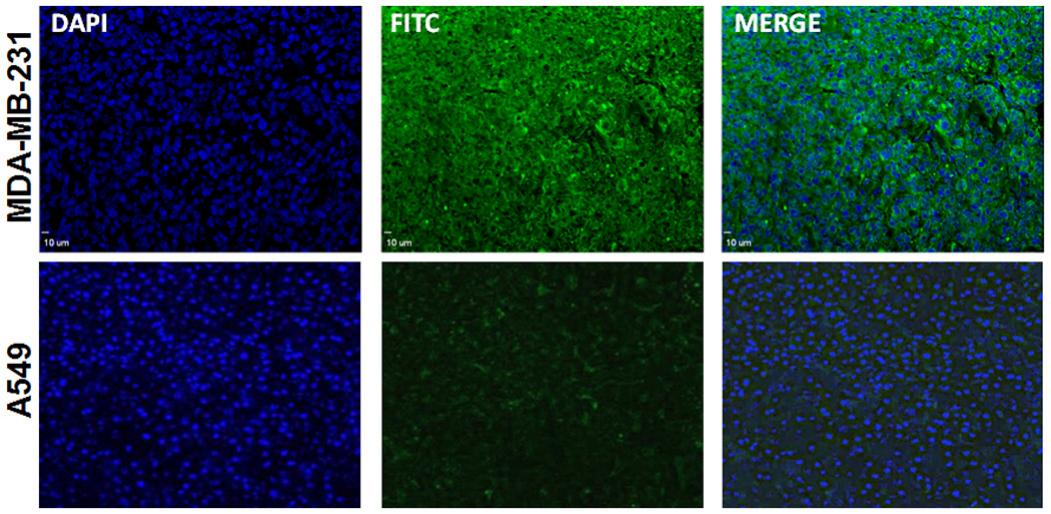
Fluorescent immunohistochemistry for MT1-MMP expression in MDA-MB-435 and A549 tumors. More MT1-MMP expressed in MDA-MB-435 tumor was observed than that of in A549 tumor. Green: FITC conjugated donkey anti-Rabbit secondary antibody. Blue: DAPI. All signals were adjusted to the same scale. Scale bar equals10 μm.

Before performing *in vivo* imaging, we also evaluated the overexpression of MT1-MMP in MDA-MB-231 tumors xenograft by immunohistochemistry. As expected, MT1-MMP was highly expressed in MDA-MB-231 tumor sections, verified by fluorescent immunostainingusing a MT1-MMP antibody (Fig 3).

### Cell fluorescence staining

**Fig 4:**
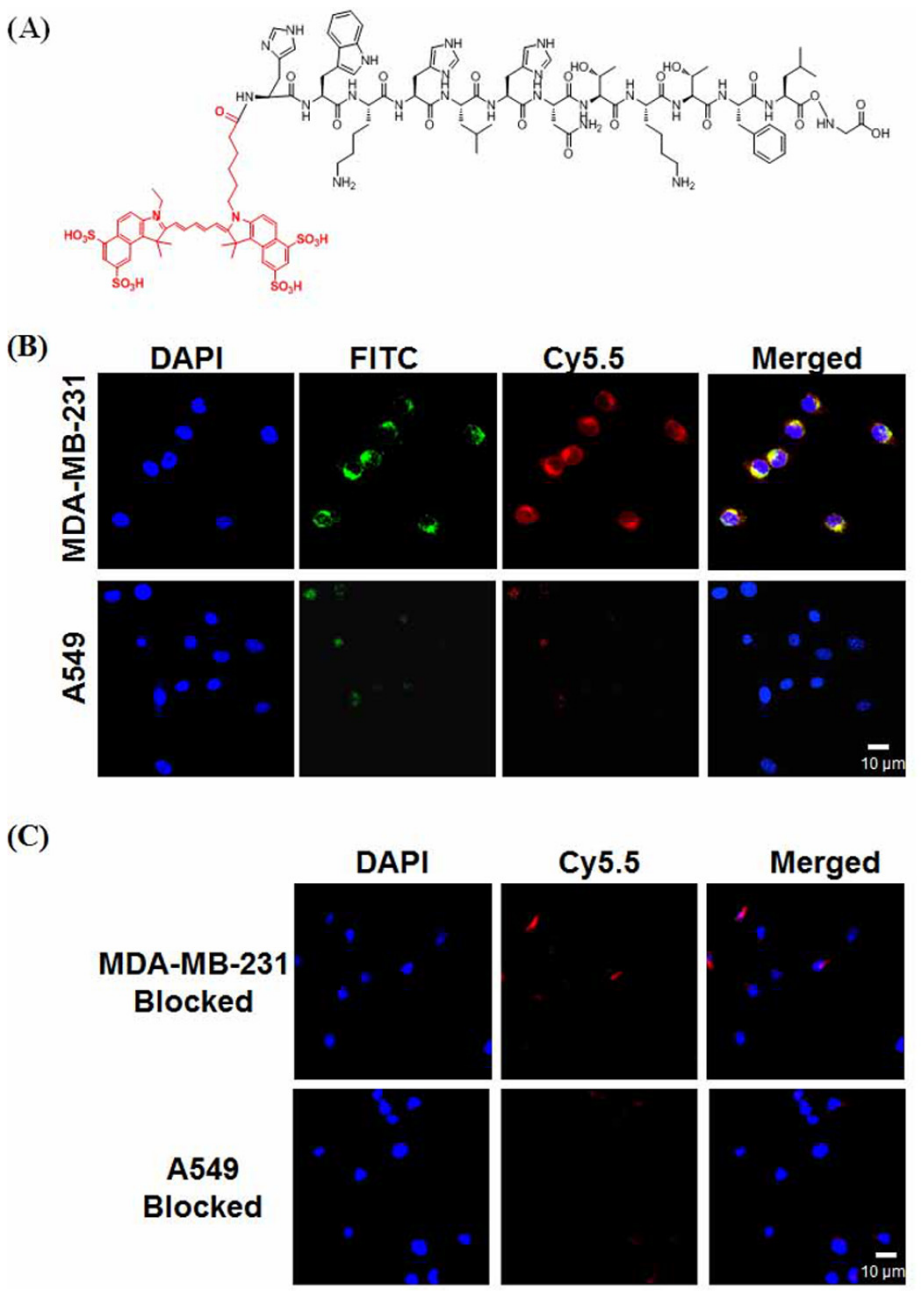
Cell labeling. (A) Chemical structure of Cy5.5 AF7p. (B) Colocalization of Cy5.5-AF7p and MT1-MMP on MDA-MB-231 and A549 cells. Red color is from Cy5.5 for Cy5.5-AF7p distribution on cellular membrane. Green color is from FITC conjugated donkey anti-rabbit secondary antibody for MT1-MMP localization. Blue color is from DAPI for nuclei visualization. Yellow color is merged color from Red and Green, indicating the colocalization of Cy5.5-AF7p and MT1-MMP. Scale bar equals 10 μm. (C). Blocking test for the specificity of Cy5.5-AF7p to MT1-MMP. Little of Cy5.5-AF7p was able to label MT1-MMP after excess amount of AF7p blocking.

**Fig 5:**
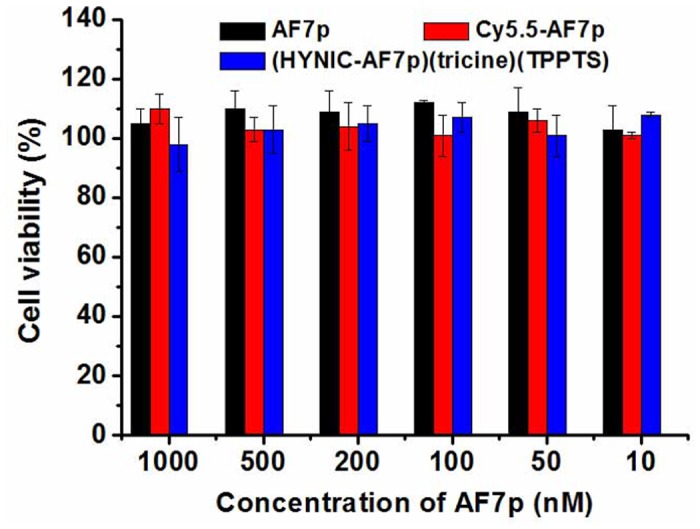
Cytotoxicity of AF7p analogs on MDA-MB-231 cells. No cytotoxicity was observed at different concentrations of AF7p, Cy5.5-AF7p and (HYNIC-AF7P)(tricine)(TPPTS).

To determine the AF7p binding specificity with MT1-MMP *in vitro*, AF7p was labeled with a near infrared dye, Cy5.5 (ex/em: 675/695 nm) for visualization of the binding of AF7p with MT1-MMP. Two cell lines with different amount of MT1-MMP expression (Fig 3) were chosen for cellular and live animal studies. Specifically, MDA-MB-231 cell that highly expresses of MT1-MMP and A549 cell which low expresses of MT1-MMP were used for investigations of AF7p specificity to MT1-MMP. Structure and characterizations of Cy5.5-AF7p were shown in S1 Fig and Fig 4A. As shown in Fig 4B, cell fluorescent staining was performed to verify the binding between Cy5.5-AF7p and MT1-MMP *in vitro*. As we expected, strong fluorescent signals from Cy5.5-AF7p was observed on MDA-MB-231 cells, while little was found in A549 cells treated with Cy5.5-AF7p. To confirm the specific binding between Cy5.5-AF7p and MT1-MMP, MT1-MMP antibody and Cy5.5-AF7p were used simultaneously for labeling of MDA-MB-231 and A549 cells. Cy5.5-AF7p and MT1-MMP was found colocalized nicely on cell membrane as shown in Fig 4. Moreover, the binding of Cy5.5-AF7p with MT1-MMP in MDA-MB-231 cells and A549 cells could be efficiently blocked when excess amount of free AF7p (100 nmol/L) was added before Cy5.5-AF7p. Little fluorescent signals were found on the MDA-MB-231 cells as shown in Fig 4C. These *in vitro* staining data support the high affinity and specificity of the identified AF7p peptide to MT1-MMP. No cytotoxicity was found for AF7p, Cy5.5-AF7p and (HYNIC-AF7P)(tricine)(TPPTS) as shown in Fig 5.

### Imaging studies

**Fig 6:**
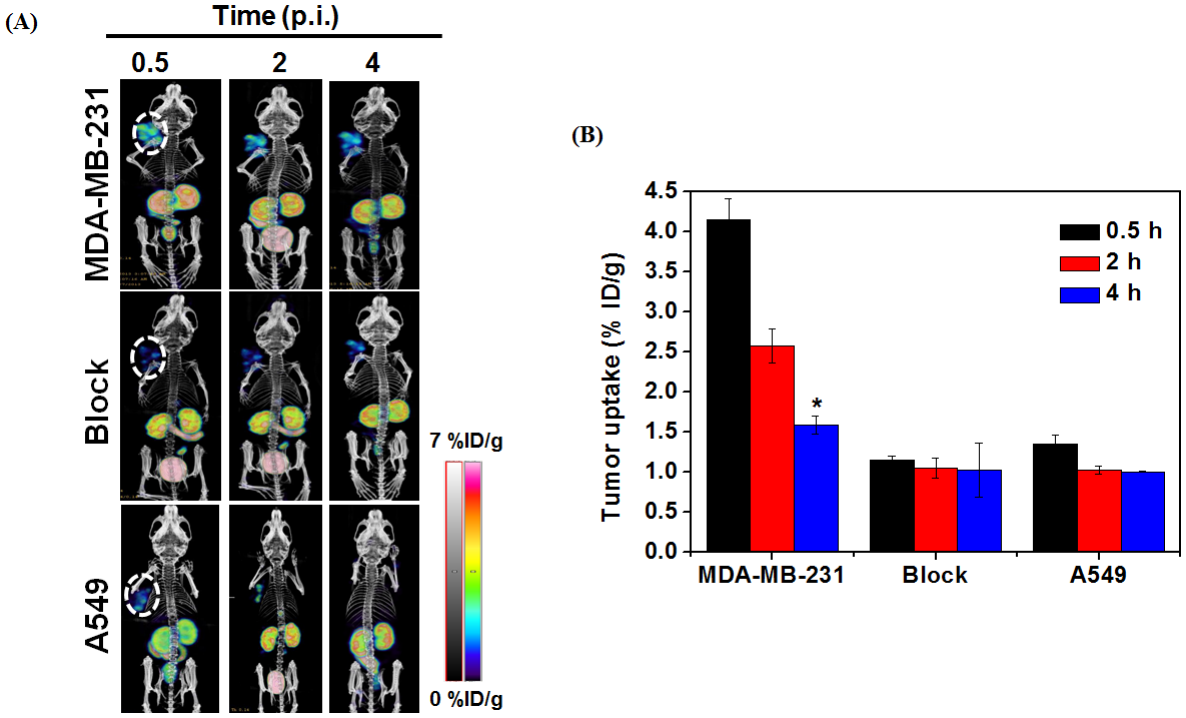
SPECT/CT images of MDA-MB-231 and A549 tumors by targeting MT1-MMP. (A) Static planar images of MDA-MB-231 tumor-bearing mice model and A549 tumor-bearing mice model after administration of 11.1 MBq/kg of [^99m^Tc](HYNIC-AF7P)(tricine)(TPPTS) at 0.5 and 2 h post injection. Excess amount (200 μg) of free AF7P was injected for blocking test. (B) Quantification of [^99m^Tc](HYNIC-AF7P)(tricine)(TPPTS) and [^99m^Tc](HYNIC-AF7P)(tricine)(TPPTS) (Blocking) in MDA-MB-231 tumor-bearing mice. Quantification of [^99m^Tc](HYNIC-AF7P)(tricine)(TPPTS) in A549 tumor-bearing mice. ROIs are shown as mean %ID/g ± SD.

To evaluate whether [^99m^Tc]-(HYNIC-AF7P)(tricine)(TPPTS) can image the MT1-MMP expression *in vivo* by SPECT, 11.1 Mbq of [^99m^Tc]-(HYNIC-AF7P)(tricine)(TPPTS) was administered intravenously into MT1-MMP-positive MDA-MB-231-tumor-bearing mice. *In vivo* imaging was performed over 4 h. Fig 6 shows representative *in vivo* whole-body images of animals at selected time points (0.5, 2 and 4 h) after injection. MT1-MMP-positive tumor region demonstrated strong signals due to the accumulations of [^99m^Tc]-(HYNIC-AF7P)(tricine)(TPPTS) with excellent tumor-to-background (4.17±0.49) at 2 hour post-injection. MT1-MMP specificity of [^99m^Tc]-(HYNIC-AF7P)(tricine)(TPPTS) accumulation was confirmed by a blocking assay, in which about 200 μg of unlabeled AF7P was injected 30 min ahead the tracer injection. As shown in Fig 6, the tumor uptake of [^99m^Tc]-(HYNIC-AF7P)(tricine)(TPPTS) was found significantly lower than unlocked tumors (1.15±0.05 vs. 4.15±0.27 at 0.5 h, 1.05±0.12 vs. 2.58±0.21 at 2 h, and 1.03±0.34 vs. 1.59±0.11 at 4 h). Very weak uptake of [^99m^Tc]-(HYNIC-AF7P)(tricine)(TPPTS) (1.35±0.11 at 0.5 h, 1.03±0.05 and 1.01± 0.00 at 4 h) was observed in A549 tumor bearing model, which expressed little MT1-MMP, suggesting that tumor uptake of [^99m^Tc]-(HYNIC-AF7P)(tricine)(TPPTS) is mediated by MT1-MMP. Taken SPECT imaging of MT1-MMP in different tumor models results, we consider AF7P is MT1-MMP dependent and specific. It should be noted that besides tumor, kidneys, and bladder also demonstrated strong signals which is consistent with *ex vivo* distribution data (Table 1), indicating that the tracer is mainly excreted through the renal-urinary routes.

**Table 1:** Biodistribution of [^99m^Tc]-(HYNIC-AF7p)(tricine)(TPPTS) in mice bearing MDA-MB-231 tumor.

Location/Time	0.5h	1h	2h	4h
**Lung**	2.11±0.06	1.45±0.18	1.01±0.03	0.70±0.09
**Heart**	2.28±0.16	1.67±0.20	0.98±0.07	0.64±0.04
**Kidney**	8.86±0.48	7.42±0.43	4.84±0.19	4.33±0.20
**Spleen**	0.90±0.07	0.71±0.06	0.56±0.06	0.41±0.02
**Liver**	5.22±0.17	3.90±0.32	2.89±0.11	2.55±0.10
**Pancreas**	0.77±0.02	0.52±0.03	0.35±0.04	0.19±0.02
**Intestine**	1.91±0.05	1.61±0.07	1.52±0.12	1.14±0.08
**Bone**	0.80±0.04	0.58±0.03	0.44±0.02	0.40±0.02
**Muscle**	0.90±0.06	0.68±0.09	0.53±0.04	0.52±0.04
**Blood**	0.36±0.02	0.28±0.01	0.20±0.01	0.19±0.01

### Biodistribution

The biodistribution study was performed using the BALB/c nude mice bearing MDA-MB-231 human breast cancer xenografts. The biodistribution of [^99m^Tc]-(HYNIC-AF7P)(tricine)(TPPTS) and tumor versus normal major organs signal ratio (T/N) for [^99m^Tc]-(HYNIC-AF7P)(tricine)(TPPTS) are summarized and listed in Tables 1 and 2, respectively. Tumor uptake of [^99m^Tc]-(HYNIC-AF7P)(tricine)(TPPTS) was 3.37 ± 0.27% ID/g, 2.59 ± 0.21% ID/g, 2.18 ± 0.11% ID/g and 1.05 ± 0.07% ID/g at indicated time points. The tumor uptake was increasing from the beginning of study and went to the highest at 2 h post injection. After 2 hours, the amount of [^99m^Tc]-(HYNIC-AF7P)(tricine)(TPPTS) steadily decreased to the end of study period. In general, [^99m^Tc]-(HYNIC-AF7P)(tricine)(TPPTS) had a rapid clearance, predominantly through the renal system at 0.5, 1, 2 and 4 hours post injection (8.86 ± 0.48%, 7.42± 0.43%, 4.84 ± 0.19% and 4.33 ± 0.20%) with minor hepatic depuration. At 2 h post injection, [^99m^Tc]-(HYNIC-AF7P)(tricine)(TPPTS) had the best T/N ratios for blood (11.11 ± 0.74), liver (0.75 ± 0.05), and muscle (4.17 ± 0.49) (Table 2).

**Table 2:** Tumor to organ ratios of [^99m^Tc]-(HYNIC-AF7p)(tricine)(TPPTS) in B mice bearing MDA-MB-231 tumor.

Location/Time	0.5h	1h	2h	4h
**Tumor/Lung**	1.60±0.18	1.81±0.34	2.17±0.14	1.51±0.30
**Tumor/Heart**	1.49±0.23	1.56±0.26	2.23±0.20	1.66±0.21
**Tumor/Kidney**	0.38±0.01	0.35±0.01	0.45±0.04	0.24±0.02
**Tumor/Spleen**	3.75±0.46	3.66±0.58	3.94±0.57	2.55±028
**Tumor/Liver**	0.65±0.06	0.67±0.07	0.75±0.05	0.41±0.04
**Tumor/Pancreas**	4.40±0.43	4.98±0.16	6.28±0.70	5.67±0.82
**Tumor/Intestine**	1.76±0.10	1.61±0.17	1.44±0.13	0.93±0.12
**Tumor/Bone**	4.21±0.20	4.50±0.51	5.01±0.38	2.60±0.15
**Tumor/Muscle**	3.77±0.42	3.86±0.75	4.17±0.49	2.02±0.09
**Tumor/Blood**	9.27±0.28	9.36±0.73	11.11±0.74	5.54±0.55

## Discussion

In this study, we used HYNIC as the BFC and tricine/TPPTS as coligands to prepare [^99m^Tc]-(HYNIC-AF7P)(tricine)(TPPTS) for *in vivo* SPECT imaging of breast cancer. The MT1-MMP targetability of AF7p was evaluated as a breast cancer SPECT imaging agent. Radiolabelling of (HYNIC-AF7P)(tricine)(TPPTS) was successful with radiochemical purity higher than 95%.

The solution stability data shows that [^99m^Tc]-(HYNIC-AF7P)(tricine)(TPPTS) is stable for 6 h in PBS and even in the presence of excess cysteine. Further evaluations showed that the kit formulation was stable, which is important for future clinical translation [14, 15]. In particular, breast tumor analysis showed good tumor uptake and T/N ratios. Based on its biodistribution and *in vivo* SPECT imaging results, [^99m^Tc]-(HYNIC-AF7P)(tricine)(TPPTS) would be useful for imaging thorax, head and neck and extremities tumors with highly expressed MT1-MMP.

We also found that [^99m^Tc]-(HYNIC-AF7P)(tricine)(TPPTS) was excreted rapidly via liver and renal route with very little radioactivity accumulation in the blood and muscle at 1 h post injection. In spite of the slow clearance from bone observed in the biodistribution data (Table 1), the images obtained at 2 h post injection showed a good tumor to background uptake ratios. This may be caused by the difference of [^99m^Tc]-(HYNIC-AF7P)(tricine)(TPPTS) injected activity between the biodistribution mice and the planar imaged mice. It is possible that increased mass of (HYNIC-AF7p) (tricine)(TPPTS) injected in the higher imaging doses could mask nonspecific binding to blood proteins and other normal tissues [15, 16]. The administration of higher doses of [^99m^Tc]-(HYNIC-AF7P)(tricine)(TPPTS) in the imaged mice appeared to achieve better tumor to normal organ ratios. These results support the use of [^99m^Tc]-(HYNIC-AF7P)(tricine)(TPPTS) for the detection of thoracic tumors, including breast cancer.

## Conclusions

[^99m^Tc]-(HYNIC-AF7P)(tricine)(TPPTS) was successfully prepared and its biodistribution and tumor imaging properties were evaluated in BALB/c nude mice bearing MDA-MB-231 human breast cancer xenograft. The relative low-cost and available supply of ^99m^Tc coupled with selective tumor uptake and rapid clearance from non-targeting organs makes it a promising agent for breast cancer detection, which remains to be validated preclinical.

## Supporting Information

S1 Fig Characterization of the purity and molecular weight of Cy5.5-AF7P by HPLC and mass spectrum. Analytical HPLC of Cy5.5-AF7P, the retention time of Cy5.5-AF7P is 11.83 min (Figure A). Molecular weight of Cy5.5-AF7P confirmed by mass spectrometer of, which is 1839.6 (Figure B).
(TIF)
